# Distinguishing Protein Corona from Nanoparticle Aggregate Formation in Complex Biological Media Using X-ray Photon Correlation Spectroscopy

**DOI:** 10.1021/acs.nanolett.4c03662

**Published:** 2024-10-01

**Authors:** Caroline E. P. Silva, Agustin S. Picco, Flavia Elisa Galdino, Mariangela de Burgos Martins de Azevedo, Marilina Cathcarth, Aline R. Passos, Mateus Borba Cardoso

**Affiliations:** † Brazilian Synchrotron Light Laboratory (LNLS), Brazilian Center for Research in Energy & Materials (CNPEM), Campinas, Sao Paulo 13083-970, Brazil; ‡ Instituto de Investigaciones Fisicoquímicas Teóricas y Aplicadas (INIFTA), Facultad de Ciencias Exactas, Universidad Nacional de La Plata - CONICET, 1900 La Plata, Argentina

**Keywords:** X-ray Photon Correlation Spectroscopy (XPCS), Silica Nanoparticles, Protein Corona, Aggregation

## Abstract

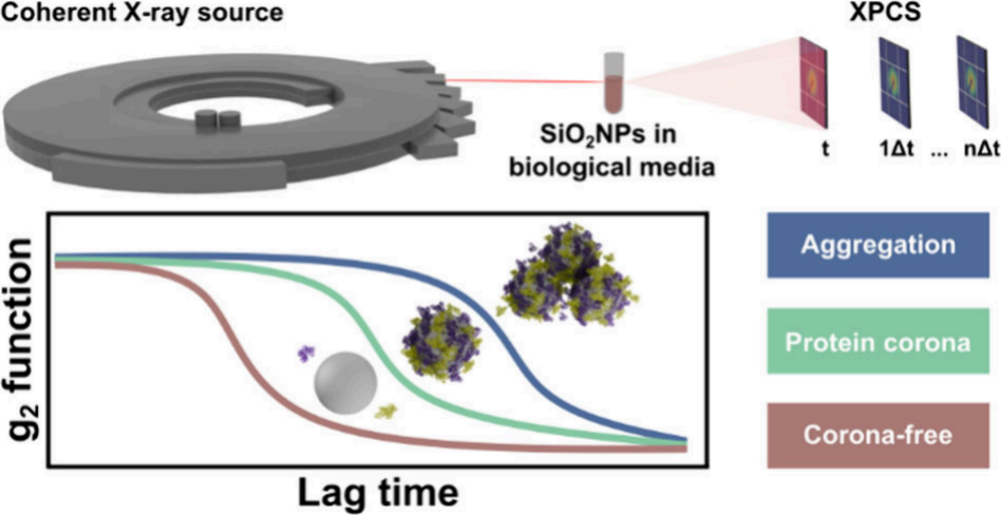

In biological systems, nanoparticles interact with biomolecules, which may undergo protein corona formation that can result in noncontrolled aggregation. Therefore, comprehending the behavior and evolution of nanoparticles in the presence of biological fluids is paramount in nanomedicine. However, traditional lab-based colloid methods characterize diluted suspensions in low-complexity media, which hinders in-depth studies in complex biological environments. Here, we apply X-ray photon correlation spectroscopy (XPCS) to investigate silica nanoparticles (SiO_2_) in various environments, ranging from low to high complex biological media. Interestingly, SiO_2_ revealed Brownian motion behavior, irrespective of the complexity of the chosen media. Moreover, the SiO_2_ surface and media composition were tailored to underline the differences between a corona-free system from protein corona and aggregates formation. Our results highlighted XPCS potential for real-time nanoparticle analysis in biological media, surpassing the limitations of conventional techniques and offering deeper insights into colloidal behavior in complex environments.

Understanding how nanoparticles (NPs) behave when exposed to biological fluids is crucial in nanomedicine. When NPs are introduced into biological systems, they interact with various biomolecules, such as proteins, forming the protein corona.^1^ This unspecific adsorption alters the biological identity of the nanoparticles and, consequently, gives rise to surfaces different from the pristine material.^2−4^ It can significantly impact the behavior, distribution and, ultimately, the NP therapeutic efficacy and safety.^5^ Furthermore, this unspecific adsorption can trigger suspension destabilization processes, resulting in the formation of aggregates or NP degradation.^6^ Consequently, it affects the diffusion, gravitational settling, and the available surface area of NPs. As a result, it induces drastic, random and unpredictable nanobio interactions, modifying biological outcomes such as cellular uptake, immune-biocompatibility, and toxicity of NPs.^7−11^ In this context, it is critical to determine the NP’s physical–chemical parameters, such as size and morphology, in complex biological environments.^12^ Fluorescence correlation spectroscopy (FCS)^13^ and dynamic light scattering (DLS)^14^ have been employed for *in situ* quantification based on diffusion coefficient measurements. However, in complex media, e.g., blood, optical detection suffers limitations since analyzed suspensions must be well diluted and transparent. Additionally, for FCS, NPs must exhibit fluorescence. Thus, these limitations prevent their *in situ* characterization of the nanobio interface in more complex and realistic biological environments.^15^

Alternatively, a very powerful technique for characterizing NPs in biological fluids, which can overcome all of the above-listed limitations, is called X-ray photon correlation spectroscopy (XPCS). XPCS is a synchrotron-based technique that uses a coherent X-ray probe to follow NPs in their native environments without perturbing their structure or function. During the XPCS experiment, a granular interference pattern, called speckle, is obtained in a two-dimensional detector reflecting the exact spatial arrangement of the objects.^16,17^ When this spatial arrangement changes as a function of time, for example, due to Brownian motion, this speckle pattern also changes, corresponding to the new arrangements formed. Consequently, these dynamics can be quantified by calculating the second-order intensity autocorrelation function *g*_2_(*q*,τ), as a function of the scattering vector *q* and the lag time τ (Section S1).^15,18,19^

Due to impressive advances in synchrotron sources and detector technologies, the life sciences community has recently started exploring XPCS as a characterization tool that allows us to go beyond those low-complexity media.^20^ Many protein-based systems, like alpha-Crystallin,^21−23^ lysozyme,^24,25^ bovine serum albumin (BSA),^26^ globulin,^27^ and prion protein,^28^ already had their dynamics investigated by this technique. In contrast, only a few XPCS studies have been reported when NPs in complex biological media are considered. The first XPCS report used complementary techniques, such as dynamic magnetic susceptibility (DMS) and rheology, to characterize the diffusion coefficients and the Brownian motion of cobalt ferrite nanoparticles coated with poly(ethylene glycol) within the synovial fluid.^29^ One year later, XPCS was employed to determine the hydrodynamic diameter (*D*_H_) of gold nanoparticles (AuNPs) across diverse media, including water, saline solutions, BSA, and blood. Interestingly, this work compared *D*_H_ values determined by XPCS with those obtained from other techniques such as DLS, electron microscopy, and small-angle X-ray scattering.^15^ Recently, the dynamics of PEGylated AuNPs within a wide range of BSA concentrations (0–265 mg/mL), compatible with the macromolecular crowding environments found in biological media, were probed.^30^ It was confirmed that the nanoparticle dynamics is affected by different BSA concentration regimes and reinforced that the presence of nanoparticles can alter the viscosity properties of dense protein solutions.

Although XPCS has proven to be a powerful tool over the past few years, the nanomedicine community still lacks relevant results from this technique. As it deals with diffusion, it is straightforward to envisage that all relevant biological events related to unspecific protein adsorption and the possible resulting aggregation can be tracked using this technique. *In situ* protein corona measurements in complex systems are of primary relevance to the nanomedicine field since most of the techniques rely on sample fractionations that perturb the nature and composition of the protein corona.^31^*In situ* analysis of corona composition, including methods like click-chemistry-based techniques^32^ and BLI-based elution^33^ for distinguishing hard and soft coronas, as well as the detailed study of their surfaces (shape, binding sites of NPs-protein, etc.), remain relevant challenges in this field.^34^ Moreover, it has also been challenging to differentiate protein corona from nanoparticle aggregate formation. Aggregates are undesirable since their inherent properties are lost due to the changes in surface energy and overall size, which give rise to unpredictable biological outcomes.^7^ Consequently, it is critical to identify protein corona and aggregates clearly while differentiating them duly.

In this work, we use XPCS to probe the dynamics of nonfunctionalized and PEGylated silica nanoparticles spanning through a range of distinct diameters. First, we used the technique to properly highlight the dynamic differences of similar particles presenting different sizes. Then, these nonfunctionalized and PEGylated particles were challenged in various environments, including aqueous and buffered solutions and media-containing proteins, aiming to gain insights into how particle size, surface functionalization, and the surrounding environment influence their diffusion properties. Our data highlight the efficacy of XPCS in distinguishing the formation of protein corona from nanoparticle aggregation in relevant biological media, which is typically not accessed by conventional characterization techniques.

Silica nanoparticles (SiO_2_) were synthesized through a modified Stöber method, as previously reported in the literature.^35−38^ This synthesis protocol allowed us to precisely size-tune the specimens for the first part of this work. Thus, the influence of the diameter on the dynamics of SiO_2_, whose diameters ranged from ∼250 to 800 nm, was investigated in an aqueous medium. Samples were named here from SiO_2_-I to SiO_2_-V as a function of nanoparticle size (Figure 1a). XPCS measurements were done at the Cateretê beamline^39^ from the Brazilian synchrotron radiation source named Sirius (Figure 1b). The parasitic scattering from the instrument was minimized to have the cleanest beam possible, which allowed us to identify subtle dynamic differences between the samples. Nanoparticle suspensions were manually injected into a capillary surrounded by vacuum to minimize the air-scattering. After each XPCS acquisition, the sample holder was extensively washed with Hellmanex 2% (v/v) and water and dried, and the capillary status was always cross-checked before the injection of the following sample. All measurements were done at room temperature, which was kept stable in the hutch during the experiments, and all colloid samples were prepared, at maximum, 30 min before the XPCS measurements.

**Figure 1:**
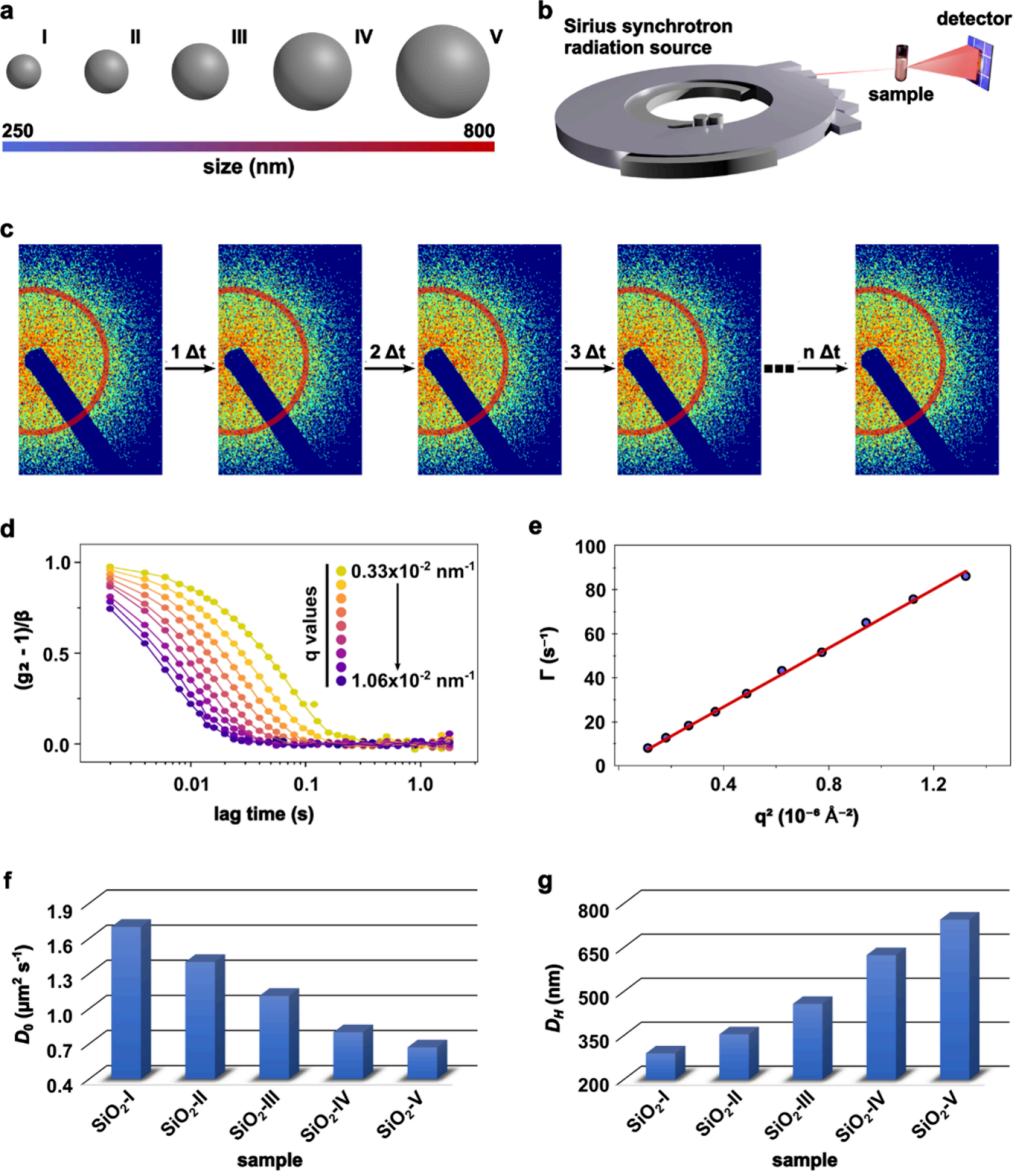
(a) Schematic representation of the silica particles of distinct sizes synthesized in this work. The particle size scale was taken into account, considering the hydrodynamic diameters that were obtained. (b) Drawing representing the X-ray photon correlation spectroscopy (XPCS) measurements highlighting the need for a synchrotron radiation source. (c) A fraction of the detector image evidencing the speckle patterns obtained during data collection. The red region represents a given *q* region that will later give rise to autocorrelation functions. (d) Normalized intensity autocorrelation functions (*g*_2_) of SiO_2_-V and (e) its corresponding relaxation rate (Γ) versus *q*^2^ data and the linear fit. (f) Diffusion coefficient (*D*_0_) and (g) hydrodynamic diameter (*D*_H_) determined for all analyzed silica particles.

As expected, all samples were colloidally stable and no signs of sedimentation were observed throughout the experiment. XPCS measurements were performed with an exposure time of 1.2 ms and a waiting time of 0.8 ms between sequential acquisitions. The detector was triggered to 500 Hz, and all measurements were completed within 2 s. No radiation damage was observed under these conditions (Section S3.4). Additionally, upon dilution, superimposable scattering curves are obtained when normalized by concentration, which eradicates multiple scattering effects.^40^ A sequence of speckle patterns was then obtained, and the resulting intensity in a given *q* region (0.33–1.06) × 10^–2^ nm^–1^ was used to generate the autocorrelation functions (Figure 1c—here we indicate a single angular range through the red area). For every angular range (*q*), the intensity obtained over time is tabulated and later converted into an autocorrelation function (*g*_2_).

The normalized *g*_2_ values plotted as a function of the lag time τ at different *q* values for all nonfunctionalized samples are shown in Figure S1. Here, we present the autocorrelation functions for SiO_2_-V with a shape typical for a colloidally stable NP (Figure 1d). By fitting these curves using the single exponential model, we obtained the relaxation time (τ) for each probed *q*. Moreover, the relation τ = Γ^–1^ allows us to obtain the corresponding relaxation rate (Γ) values. As expected, all KWW exponents (γ) obtained by the beamline software fittings^41^ were close to 1 (Table S2) and all the curves Γ vs *q*^2^ presented a linear-like shape (Figure 1e and Figure S2). These are two strong indications that all the NPs displayed Brownian diffusion, agreeing well with what has been reported in the earlier literature.^15,29,30^Equation S4 was then applied to obtain the *D*_0_ (diffusion coefficient) value, using a linear fit whose slope generated *D*_0_ to each system (Figure 1f). As expected, a clear decrease in *D*_0_ was seen when the nanoparticle size was increased. The Stokes–Einstein equation (eq S5) was then applied to determine *D*_H_ for each derived *D*_0_ (Figure 1g and Table S2). As expected, these values are inversely proportional to the *D*_0_ values since all particles were measured in the same medium.

Subsequently, SiO_2_ nanoparticles, very similar to the SiO_2_-I one, were synthesized and functionalized with 2000 g/mol polyethylene glycol groups (PEG-SiO_2_) (Section S2.3) and the dynamic properties of these two particles were then challenged in more complex environments (Figure 2a). These NPs were kept in a fixed final concentration (10 mg/mL) in phosphate-buffered saline (PBS 10 mM) with the possibility of media supplementation depending on the experiment. For the specific purpose of this work, the medium was supplemented with either bovine serum albumin (BSA; 5 mg/mL) or fetal bovine serum (FBS; 10% v/v). In addition, all samples prepared for this part of the work were dispersed in nonsupplemented or supplemented PBS and immediately loaded into the capillary for XPCS measurements (Section S2.5). As a general trend, all of the obtained correlation curves for SiO_2_-I and PEG-SiO_2_ resemble those presented in Figure 1d. Furthermore, these systems displayed typical Brownian behavior, regardless of functionalization or media employed (Section S3.2).

**Figure 2:**
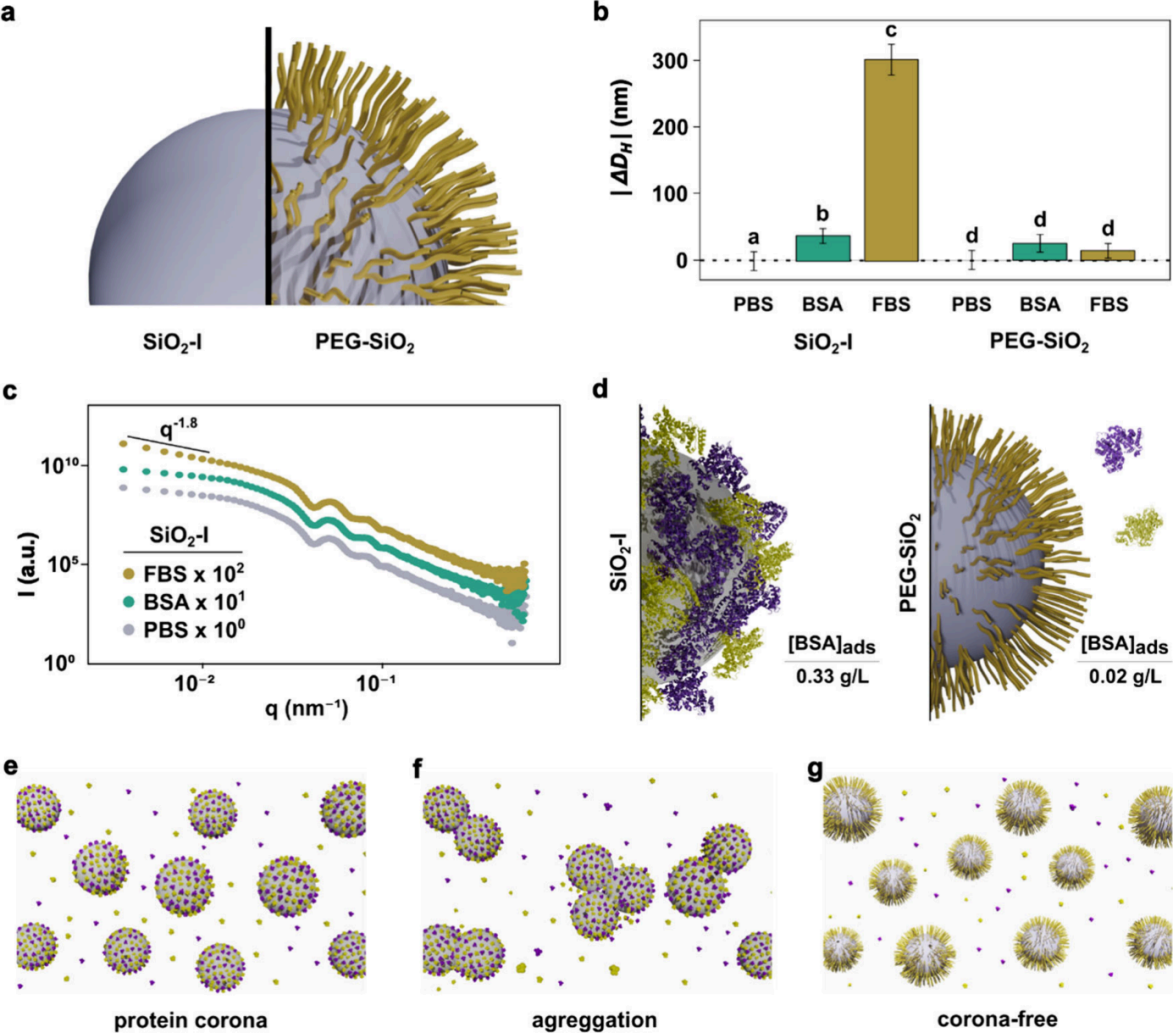
(a) Schematic representation of nanoparticles used in the second part of this work, bare SiO_2_-I and PEG-SiO_2_. (b) Absolute value of the variation in hydrodynamic diameter (|Δ*D*_H_|) of SiO_2_-I and PEG-SiO_2_ in PBS, BSA, and FBS media. Results are displayed as mean ± standard deviation. One-way analysis of variance (ANOVA) with Tukey’s test were used for data statistical analysis. Differences were considered significant when *P* < 0.05. Bars labeled with the same letter indicate no significant difference, while bars labeled with different letters represent statistically significant differences. (c) SAXS curves of bare SiO_2_-I in PBS, BSA, and FBS media. (d) Comparison between the concentration of BSA in the precipitated ([BSA]_ads_) obtained by BCA assay for SiO_2_-I and PEG-SiO_2_. Schematic representation of (e) protein corona formation on the surface of bare SiO_2_-I. (f) Aggregation of bare SiO_2_-I and the (g) corona-free effect of PEG-SiO_2_.

Figure 2b shows a comparative analysis of the |Δ*D*_H_| values for the nonfunctionalized and PEGylated SiO_2_ in PBS 10 mM, BSA 5 mg/mL (in PBS 10 mM), and FBS 10% (in PBS 10 mM). These values were obtained by subtracting D_H_ of PBS medium from the D_H_ of the studied medium. A one-way analysis of variance (ANOVA) was performed to determine if there were significant differences within each group (Section S3.3). Two distinctly different scenarios were observed. For the unmodified SiO_2_, we observed a notable trend where an increase in the complexity of the protein-rich medium corresponded to an increased |Δ*D*_H_|. Statistically, the |Δ*D*_H_| values showed significant differences, specifically attributed to the protein corona (in BSA) and aggregation (in FBS). In the latter, the overall size of the aggregates cannot be taken as an absolute value rather than an aggregation indication. In contrast, PEG-SiO_2_ remained nearly unaffected by the presence of proteins, as indicated by the lack of significant variation in their |Δ*D*_H_| values based on the ANOVA tests. PEGylated nanoparticles are known for preventing the formation of a protein corona. This is achieved through functional groups that create a hydration shell around the nanoparticles, effectively preventing nonspecific protein adsorption.^42−44^ PEG functionalization also enhances colloidal stability through strong hydration, as PEG’s hydrophilic nature forms hydrogen bonds with water molecules.^7^ At optimal grafting densities, PEG creates steric repulsion, preventing nanoparticle aggregation.^45−47^ This effect is evident in our observations, where PEGylation helped to maintain a stable dispersion of nanoparticles.

Although it is considerably straightforward to understand the PEGylated particle’s noninteracting behavior, the |Δ*D*_H_| increase for the nonfunctionalized counterpart can be interpreted based on two possible scenarios. The first and most intuitive method relies on attributing such |Δ*D*_H_| differences to viscosity changes. However, this scenario cannot explain how the PEGylated structures kept the |Δ*D*_H_| values almost constant across distinct complex media. In addition, although there are differences in the viscosity between these used media, the viscosity measured values and the observed differences are insufficient to ascribe such significant |Δ*D*_H_| differences. Section S5 details the viscosity values for various media. While these values show slight deviations from those reported in the literature,^48,49^ these discrepancies did not affect the observed trends in our results since the differences between the media viscosity values are minimal. In contrast, the impact of the protein media on *D*_0_ variation (Table S3) is significant, playing a central role in the observed changes in the calculated |Δ*D*_H_| values.

Alternatively, we can assume that the |Δ*D*_H_| changes can be attributed to a structural evolution due to the possible interaction between nonfunctionalized SiO_2_ and proteins. Considering this scenario, the most reasonable explanation for the |Δ*D*_H_| changes relies on a natural evolution that evolves to protein corona formation (media supplemented with BSA), which later undergoes aggregation in a more complex environment (media supplemented with FBS). Similar trends have already been reported in the literature, suggesting that SiO_2_ tends to form a protein corona in BSA-supplemented media, while aggregation is often seen in the presence of FBS at a much lower NP concentration range.^14,50^ However, the |Δ*D*_H_| value exceeds tens of nanometers for SiO_2_-I in BSA and could also suggest nanoparticle aggregation. In parallel, a few literature reports have described thicker protein coronas for distinct conditions.^51,52^ Although there is a vigorous debate about this topic in the literature, the corona size found in our work appears reasonable, given that we are not working with a diluted model system.^53^ The corona formation for SiO_2_-I in BSA is further confirmed by the SAXS analyses presented below.

In this work, we recognize that XPCS has been used for the first time to address protein corona formation and aggregation. Consequently, we decided to use standard-like techniques to cross-check the scenario described above. Initially, synchrotron small-angle X-ray scattering (SAXS) was used to investigate the static nature of these nonfunctionalized samples in different environments. Figure 2c presents SAXS data for SiO_2_-I in pure PBS as well as when the medium was supplemented with either BSA or FBS. No significant difference exists between the SAXS curves of nonsupplemented medium and that containing BSA, reinforcing no aggregation for these systems. Although protein corona has been reported by SAXS, we understand that the scattering power, due to the highly concentrated suspension and the large NP size, prevents us from clearly identifying protein corona by SAXS. On the other hand, meaningful SAXS profile changes can be seen when the sample supplemented with FBS is compared to its counterparts and is related to the formation of aggregates. In the low-*q* part (*q* < 0.02 nm^–1^), the scattering profile is due to the presence of individual structures or the formation of aggregates, which induces an evident change of the scattering curve. For the sample in the presence of FBS, the inclination in the low-*q* region was considerably changed, and the power law decay indicates aggregate formation. These experimental SAXS data were deconvoluted by considering two different scattering contributions: (a) SiO_2_ (represented by polydisperse spheres) and (b) aggregates fitted using a power-law decay (*P*) together with a cutoff constrained to the radius of SiO_2_, as previously reported in the literature (Section S6).^50,54^ The fitting provided a Porod decay exponent (*P*) value of 1.81 (Figure S8), which agrees well with the ones obtained for smaller bare SiO_2_ (*D* ≅ 30 nm) dispersed in PBS media supplemented with FBS.^50^ SAXS measurements were also conducted on the PEG-SiO_2_ samples. The resulting curves indicated high colloidal stability, as no signs of aggregation were observed, regardless of the media used (Figure S9).

Although SAXS efficiently discriminates aggregates of all other sample states, protein corona confirmation was still missing. DLS measurements were not performed to confirm the protein corona formation, as DLS is limited to dilute suspensions. Consequently, this technique is inadequate to probe a 10 mg/mL SiO_2_-I suspension. So, we challenged our particles through a BCA (bicinchoninic acid) assay (Section S2.8). In this test, the protein concentration in a sample is determined via a colorimetric method based on the ability of proteins to reduce copper ions Cu^2+^ to Cu^+^ under alkaline conditions. This reduction forms a purple complex that can be detected spectrophotometrically. The intensity of the color is directly proportional to the protein concentration in the sample, allowing for accurate protein quantification.^55^ Particles were incubated at 10 mg/mL in PBS supplemented with BSA, centrifuged, and resuspended, and the BCA measurements were done. The result clearly indicates that a protein corona is formed when these nonfunctionalized particles are incubated with BSA (Figure 2d), with a BSA adsorbed concentration ([BSA]_ads_) of 0.33 g/L. Moreover, we estimated the concentration of a monolayer of BSA on SiO_2_-I using geometric considerations and calculated a value of 0.37 g/L (Section S8). Remarkably, this estimate closely aligns with the experimentally determined result, even though it was derived using a geometric approximation, fundamentally different from the BCA assay, a spectrophotometric-based technique. On the other hand, PEG-SiO_2_ presented just a small fraction of adsorbed protein ([BSA]_ads_ = 0.02 g/L), corroborating the corona-free effect induced by the PEG functional groups. BCA and SAXS agree well with XPCS data, which promises groundbreaking insights into protein–nanoparticle interactions, vital for advancing nanoparticle studies in biological systems and nanomedicine. However, challenges remain, such as data processing pipelines, to handle vast data volumes. Developing viscosity databases for biological media, such as serum and cell cultures, is also crucial. As the XPCS community grows, these challenges are expected to be addressed, positioning the technique as a leading tool for exploring the nanobio interface, enhancing our understanding of nanoparticle behavior, and unlocking their potential as nanomedicines.

In summary, we present a successful demonstration of XPCS in monitoring the dynamics of silica nanoparticles, highlighting its efficacy in capturing changes induced by variations in the diameter or exposure to complex biological environments. The diffusion coefficients were accurately determined using the single exponential model to fit the autocorrelation curves, and regardless of diameter, the complexity of the biological media, or the presence/absence of PEG functionalization, all the silica nanoparticles exhibited Brownian motion dynamics. Using the Stokes–Einstein equation, we successfully obtained hydrodynamic diameters across the entire range of nanoparticle sizes used in this study. XPCS effectively discerned *in situ* variations in the diffusion of bare silica nanoparticles induced by protein corona formation and aggregation, as well as discriminated the noninteracting nature of proteins and the PEGylated silica nanoparticles. All experiments were done in highly concentrated and complex media, without dilution, fractionation, or any other alteration, a feat not achievable by other techniques.

## Supporting Information Available

Theoretical considerations regarding XPCS analysis, materials and methods, XPCS results, STEM images of the bare and PEGylated SiO_2_, viscosity measurements, modeling of the SAXS curve of SiO_2_-I in FBS, SAXS curves of PEG-SiO_2_ in different media, and theoretical estimation of the concentration of adsorbed BSA (PDF)
