# Supplementary material for: Distinguishing Protein Corona from Nanoparticle Aggregate Formation in Complex Biological Media Using X-ray Photon Correlation Spectroscopy
Source: Nano Lett. 2024 Oct 1;24(42):13293–9. doi: 10.1021/acs.nanolett.4c03662 (PMC11505373; doi:10.1021/acs.nanolett.4c03662)
Supplement: Supplementary file 1 — nl4c03662_si_001.pdf [file nl4c03662_si_001.pdf]

Supporting Information

Distinguishing Protein Corona from Nanoparticle Aggregates Formation in Complex Biological Media Using X-Ray Photon Correlation Spectroscopy

Caroline E. P. Silva[1], Agustin S. Picco[2], Flavia Elisa Galdino[1], Mariangela de Burgos Martins de Azevedo[1], Marilina Cathcarth[2], Aline R. Passos[1], Mateus Borba Cardoso[1*]

[1] Brazilian Synchrotron Light Laboratory (LNLS), Brazilian Center for Research in Energy & Materials (CNPEM), Campinas, Sao Paulo, 13083-970, Brazil.

[2] Instituto de Investigaciones Fisicoquímicas Teóricas y Aplicadas (INIFTA), Facultad de Ciencias Exactas, Universidad Nacional de La Plata - CONICET, 1900 La Plata, Argentina.

* Corresponding author: cardosomb@lnls.br

Contents

Section S1 – Theoretical considerations regarding XPCS analysis 3

Section S2 – Materials and Methods 5

2.1. Materials 5

2.2. Synthesis of bare silica nanoparticles 5

2.3. Functionalization of bare SiO_2 nanoparticles with silanized PEG_{2000} 8

2.4. Nanoparticle characterization 8

2.5. XPCS experiments 9

2.6. XPCS data processing 9

2.7. SAXS measurements 11

2.8. Protein corona experiments 12

Section S3 – XPCS results 13

3.1. Effect of diameter 13

3.2. Effect of media 17

3.3. One-way analysis of variance (ANOVA) 21

3.4. Damage radiation tests 22

Section S4 – STEM images of the bare and PEGylated SiO_2 24

Section S5 – Viscosity measurements 25

Section S6 – Modeling of the SAXS curve of SiO_2-I in FBS 26

Section S7 - SAXS curves of PEG-SiO_2 in different media 28

Section S8 – Theoretical Estimation of the Concentration of Adsorbed BSA 30

References 32

Section S1 – Theoretical considerations regarding XPCS analysis

XPCS acts as a space-time resolved X-ray probe that measures dynamics at the nanometer scale. When exposing a system of scatterers (nanoparticles, for example) to a coherent beam of X-rays, a granular interference pattern is obtained in the two-dimensional detector, called speckle, which reflects the exact spatial arrangement of the scattering objects. If the spatial arrangement of these scatterers undergoes changes as a function of time, for example, due to the Brownian motion, this speckle pattern also changes, correspondingly to the new arrangements that are formed.[1–3] The correlation between series of two-dimensional speckle patterns is determined by the second-order intensity autocorrelation function, $g_2(q,t)$, calculated for each pixel of the detector area according to the relationship:

$$g_2(q,t) = \frac{\langle I(q,t_0)\, I(q,t_0+t)\rangle}{\langle I(q)\rangle^2} \text{ (Equation S1)}$$

where $I(q,t_0)$ and $I(q,\ t_0+t)$ are the intensities at a given pixel measured at time t_0 and $t_0 + t$, respectively, and the square brackets indicate an average over time and over the pixels referring to the same module of the scattering vector q. The t interval is defined by the detector frame rate.[2,4] The function g_2 is associated with the electric field–field autocorrelation function $g_1(q,t)$ (also known as first-order correlation function or intermediate scattering function) through the Siegert relation:

$$g_2(q,t) = 1 + \beta|g_1(q,t)|^2 \text{ (Equation S2)}$$

β represents the contrast factor (also known as Siegert factor), an instrumental parameter referring to optical contrast and directly related to the degree of coherence of the X-ray beam.[1,2]

The function $g_2(q,t)$ can be modeled using the Kohlrausch–Williams–Watts (KWW) function (a stretched exponential):[5]

$$g_2(q,t) = \beta e^{(-2\Gamma(q)t)^{\gamma}} + 1 \text{ (Equation S3)}$$

where $\Gamma(q)$ is the relaxation rate and γ is the KWW exponent. Γ is related to the relaxation time (τ) as $\tau = \Gamma^{-1}$. On the other hand, γ is a measure of the distribution of relaxation times of individual particles. If $\gamma = 1$, KWW equation represents a single exponential decay. If $\gamma \neq 1$, the system exhibits a stretched exponential behavior, indicating a distribution of relaxation times.

Ideally, the term "+1" in Equation S3 should accurately reflect the long-time value of $g_2(t)$. However, due to experimental noise and background interference, this baseline can deviate slightly from unity. To account for these practical deviations, the equation is often modified to include a baseline factor B: $g_2(t) = \beta e^{(-2\Gamma t)^{\gamma}} + B$.

The relaxation rate (Γ) displays a characteristic $\vec{q}$-dependence $\Gamma \propto q^p$. Both exponents, p and γ, specify the type of the dynamics related to a system. For dynamics dominated by Brownian diffusion, γ = 1 and p = 2:[5]

$$\Gamma(q) = D(q) \cdot q^2 \text{ (Equation S4)}$$

In this case, $D(q) = D_0$ (free diffusion coefficient) on all length scales, allowing access to the dynamic properties of NPs. Furthermore, from D_0, it is possible to calculate the hydrodynamic diameter (D_h) of nanoparticles using the Stokes-Einstein equation:

$$D_0 = \frac{k_B T}{3\pi\eta D_h} \text{ (Equation S5)}$$

where k_B is the Boltzmann constant, T is the temperature and η is the dynamic viscosity of the solvent in which they are dispersed.[1,5]

Values of the D_0 and D_H obtained for SiO_2-I to -V samples applying single exponential model, as well as KWW exponents (γ) are shown in this document, in the Table S2 (Section S3).

Section S2 – Materials and Methods

2.1. Materials

Tetraethyl orthosilicate (TEOS), ammonium hydroxide (28-30%), anhydrous ethyl acetate, 1,3-propanesultone, N,N-dimethyl-3-aminopropyltrimethoxysilane (DMAPTMS), polyethylene glycol methyl ether (M_W: 2000 g mol^{-1}), triethylamine, 3-(triethoxysilyl) propyl isocyanate, phosphate buffer saline and bovine serum albumin, all from Sigma-Aldrich, were used without further purification. Fetal bovine serum (FBS) and ethanol P.A. were purchased from Gibco and Merck, respectively. All the solutions and suspensions were dispersed in ultrapure water obtained from a water purification system (Purelab from ELGA - resistivity of 18.2 MΩ·cm).

2.2. Synthesis of bare silica nanoparticles

A set of silica nanoparticles (SiO_2) were synthesized employing the Stöber method with few modifications, varying the volume of ammonia employed in the synthesis.[6–8] 15 mL of ethanol was mixed with an aliquot of ammonium hydroxide solution (28-30%) in an Erlenmeyer flask. After 30 min, two 0.4 mL aliquots of TEOS were added to the reaction with a time spacing of 30 minutes between the two additions. Four different volumes of ammonium hydroxide solution were used, with the aim of synthesizing SiO_2 nanoparticles that varied in size: 1.3 mL (SiO_2-II); 1.4 mL (SiO_2-III); 1.8 mL (SiO_2-IV) and 2.2 mL (SiO_2-V) (Table S1). After adding the last aliquot of TEOS, the reaction was kept sealed and under constant stirring at room temperature for 24 hours. The purification of these nanoparticles was carried out by centrifugation with ethanol and another five centrifugations with ultrapure water, for 10 minutes at 10,000 rpm each cycle, to reach a

pH close to ~6. After all these steps, the SiO_2 nanoparticles were kept suspended in water at 10-12°C.

Another set of silica nanoparticles (SiO_2-I) were synthesized based on an adapted protocol of Kim and collaborators.[9] 5 mL of ammonium hydroxide solution (28-30%), 80 mL of ethanol, and 10 mL of ultrapure water were mixed in a round bottom flask, under magnetic stirring, for 30 minutes. After this time, 10 mL of TEOS was added to the reaction system and the chamber was kept sealed at room temperature for two hours (Table S1). The purification and storage of these nanoparticles were performed as already described for the first set of nanoparticles.

Table S1. Description of the reactional conditions to synthesize the bare silica nanoparticles investigated in this work:

Sample	Ethanol (mL)	Water (mL)	Ammonia (mL)	TEOS (mL)	Time (h)	Temperature (ºC)
**SiO_2-I**	80	10	5.0	10	2	25
**SiO_2-II**	15	0	1.3	0.8	24	25
**SiO_2-III**	15	0	1.4	0.8	24	25
**SiO_2-IV**	15	0	1.8	0.8	24	25
**SiO_2-V**	15	0	2.2	0.8	24	25

2.3. Functionalization of bare SiO_2 nanoparticles with silanized PEG_{2000}

Another batch of bare SiO_2 nanoparticles was prepared and purified, like the protocol of SiO_2-I described above. The first step of the PEGylation was to prepare the silanized PEG_{2000} (PEG_{2000}-TESPI). 1.476 g of polyethylene glycol methyl ether (PEG_{2000}) was added to a 25 mL two-necked sealed flask, which was kept under vacuum at 40°C for 16 hours. Then, the vacuum was interrupted, and the system was heated to 80°C under argon atmosphere. Subsequently, triethylamine (0.02 mL) was added to the reaction flask followed by the addition of 3-(triethoxysilyl) propyl isocyanate (0.2 mL). After 8 hours of reaction, the argon flow was stopped, and the reaction was stirred for another 20 hours. The product obtained was used without purification in the second step of the synthesis. Firstly, 1.358g of PEG_{2000}-TESPI was vortexed in 6.5 mL of ultrapure water in a vial for few seconds and then added to a flask containing 80 mL of bare SiO_2 nanoparticles and 2.6 mL of ammonium hydroxide solution (28-30%). The reaction was kept under magnetic stirring at 25°C for 16 hours, and posteriorly heated for 4 hours at 80°C. Finally, the suspension of PEG-SiO_2 was purified by 4 cycles of centrifugation in ultrapure water for 10 minutes at 12,000 rpm. After all these steps, the aqueous suspensions of PEG-SiO_2 were kept stored at 10-12°C.

2.4. Nanoparticle characterization

After each synthesis, the hydrodynamic diameter ($D_{H,DLS}$) of each sample was measured using a Malvern Zetasizer ZS equipment (Malvern Instruments Ltd., UK – detection angle of 173° and laser wavelength of 633 nm).

The morphology of SiO_2-I and PEG-SiO_2 were evaluated by scanning transmission electron microscopy (STEM), where images were acquired using a SEM-

FEI Inspect F50 microscope, operating at 30 kV. Five μl of the sample was placed on a carbon grid and the solvent was evaporated for 10 minutes, with the excess removed using filter paper. The analyzes were carried out at the National Nanotechnology Laboratory (LNNano, Campinas, Brazil).

2.5. XPCS experiments

Figure 1a schematizes the synchrotron-based small-angle X-ray photon correlation spectroscopy (XPCS) experiment used during this work. XPCS measurements were performed at the Coherent X-ray Scattering beamline (Cateretê) at the Brazilian Synchrotron Light (LNLS) facility, in a SAXS geometry with a coherent 9 keV X-ray beam of 40 x 40 μm^2 size focused on the sample (wavelength λ = 1.38 Å). The scattering patterns were recorded using a PIMEGA 540D detector located inside a vacuum chamber 15.04 m downstream of the sample position, covering a q range of 0.00334-0.0115 nm^{-1}, where q is the magnitude of the scattering vector given by $\left(\frac{4\pi}{\lambda}\right) sin\left(\frac{\theta}{2}\right)$, and θ is the scattering angle. The sample was filled into 1.5 mm diameter quartz capillaries, the capillaries were placed in a sample cell in vacuum at 25 °C. A total of 1000 images containing the speckle patterns were collected in a frame rate of 500 Hz for each sample (exposure time = 0.0012 s and acquisition time = 0.002 s). The processing of XPCS data during the beamtime was performed using a Python software package.[10]

2.6. XPCS data processing

To analyze the XPCS data, we developed a Python script that automates the data processing steps, ensuring a consistent and efficient workflow. The script is organized in the Jupyter Notebook 'hdf5_xpcs_fitlab.ipynb' and the accompanying Python module

'XPCS_functions.py', available in a GitHub repository. This module contains essential functions for handling XPCS data and performing the necessary analyses. The script performs the following tasks:

Data Loading: The script reads XPCS data from HDF5 files using the *h5py* library. This format is commonly used for storing large amounts of scientific data. The input data includes precomputed second-order intensity autocorrelation functions, $g_2(t)$, for various values of the scattering vector q, provided by the synchrotron facility after radial integration of the speckle images.

Data Structuring: Upon loading the data, the script organizes it into a *Pandas* DataFrame. Each DataFrame corresponds to an HDF5 file, with the first column representing time t and subsequent columns representing g_2 values for different q values.

Curve Fitting: By plotting the g_2 function against time t, the script fits the data to the single exponential model and reports the parameters for it, along with the R-squared (R^2) value:

- Single Exponential: $g_2(t) = A + Be^{-2Ct}$

The fitting is performed using nonlinear least squares optimization. In this model, the script extracts and reports parameters such as the baseline (A), β (B) and relaxation rate (C). Additionally, the R-squared (R^2) value is calculated using the *r2_score* function from the *sklearn.metrics* module, which measures the proportion of variance in the dependent variable that is predictable from the independent variables. An R^2 value close to 1 indicates a good fit, whereas a value closer to 0 indicates a poor fit.

Diffusion Coefficient Calculation: The script calculates the diffusion coefficient (D_0) in two ways:

1. Direct Calculation: Using the fitted parameters from the previous models, the script calculates D_0 for each q value using the Equation S4: $D_0 = C/q^2$. Only the fits with R^2 greater than 0.9 are considered, and the diffusion coefficients for these fits are averaged to obtain a final value.

2. Linear Fit: The script plots the relaxation rate (C or C_1) versus q^2 and performs a linear fit. The slope of the linear fit gives the diffusion coefficient.

2.7. SAXS measurements

SAXS experiments were also conducted at the Cateretê beamline, within the LNLS facility, utilizing a focused coherent 9 keV X-ray beam and a wavelength (λ) of 1.38 Å. The scattering data was captured employing a PIMEGA 540D detector housed within a vacuum chamber positioned 15.04 m downstream from the sample, enabling coverage of a q-vector range spanning from 0.00362 to 0.63751 nm^{-1}. The analyzed samples pertained to the second part of the study, delving into the impact of medium (PBS 10 mM, BSA 5 mg/mL and FBS 10%) and functionalization in mixtures involving SiO_2-I and PEG-SiO_2 (both 10 mg/mL). In order to verify if there were multiple scattering effects in our curves, we diluted nanoparticles' suspensions and superimposable SAXS curves were obtained when normalized by concentration. Most importantly, as shown by Semeraro et al.[11], working with silica nanoparticles of approximately 450 nm, multiple scattering effects have a negligible influence on XPCS data, which is the focus of our study.

2.8. Protein corona experiments

Protein corona formation was investigated by bicinchoninic acid (BCA) assay. Mixtures formed by 10 mg/mL SiO_2-I or PEG-SiO_2 and BSA 5 mg/mL, all of them dispersed in PBS 10 mM, were centrifuged and washed three times and the concentration of BSA in the final precipitated were quantified by BCA assay. Firstly, a calibration curve of BSA were determined, within a range of 0 to 2 mg/mL. Then, the precipitates obtained from the mixtures were dispersed in PBS 10 mM and analyzed based on the previous calibration curve. Both the curve and sample points were mixed with the BCA working reagent provided by a ThermoFisher Scientific® BCA protein assay kit. Each point was placed in a 96-well plate and incubated at 37°C in a ThermoMixer C (Eppendorf ®). Then, the absorbance of each well was read in a Thermo Scientific® Varioskan LUX Multimode Microplate Reader in a fixed wavelength of λ = 562 nm. Both pure suspensions of SiO_2-I or PEG-SiO_2, without BSA, also presented an absorbance in this λ, which was discounted in the obtained absorbance in order to calculate only the BSA concentration in the precipitate.

Section S3 – XPCS results

3.1. Effect of diameter

The autocorrelation functions g_2 plotted as a function of the lag time τ at different q values obtained by the samples SiO_2-I, II, III and IV, all dispersed in ultrapure water, were shown in the Figures S1(a-d). By fitting these curves using the single exponential model, we accessed the relaxation time (τ) in each q probed, and we applied the relation $\tau = \Gamma^{-1}$ to obtain the corresponding relaxation rate (Γ) values. As all KWW exponents (γ) obtained by the beamline fittings were close to 1 and all the curves Γ vs. q^2 (Table S2 and Figures S2(a-d)) presented a linear shape, we have two strong indications that all the nanoparticles displayed Brownian motion dynamics. Then, Equation S4 was applied to find the D_0 (diffusion coefficient), using a linear fit whose slope provided D_0 to each system. From each D_0, we calculated $D_{H,XPCS}$ using the Stokes-Einstein equation (Eq. S5).

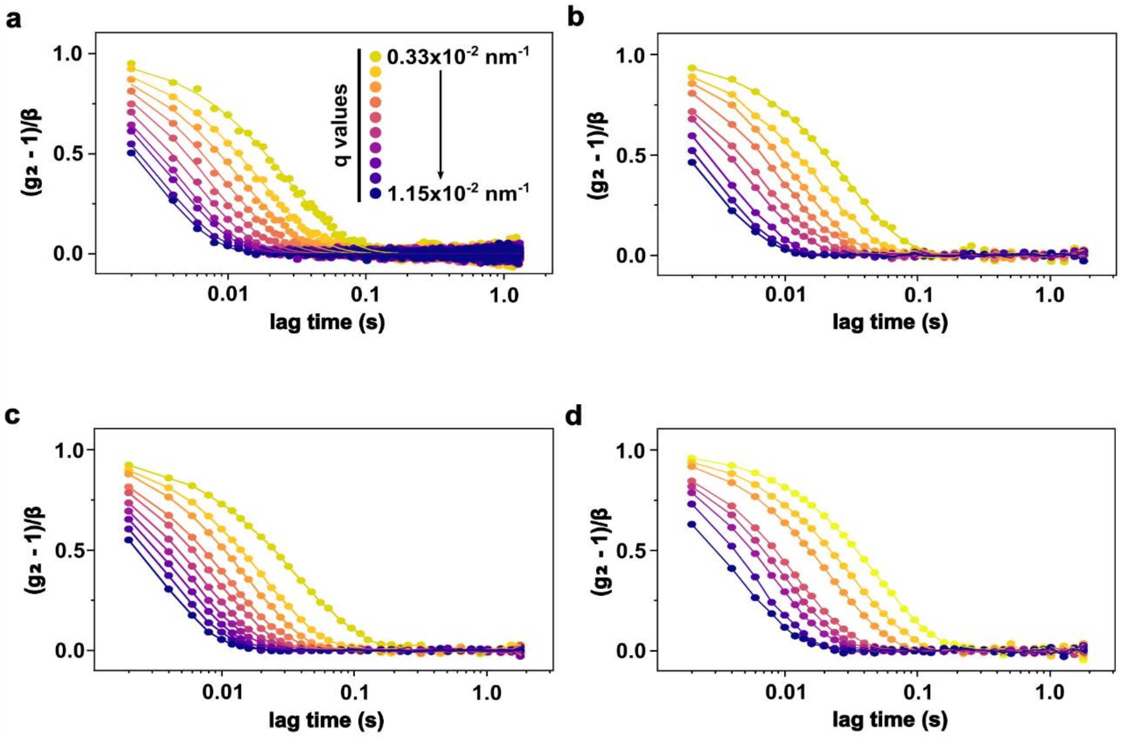

Figure S1. Normalized intensity autocorrelation functions of (a) SiO_2-I, (b) SiO_2-II, (c) SiO_2-III and (d) SiO_2-IV.

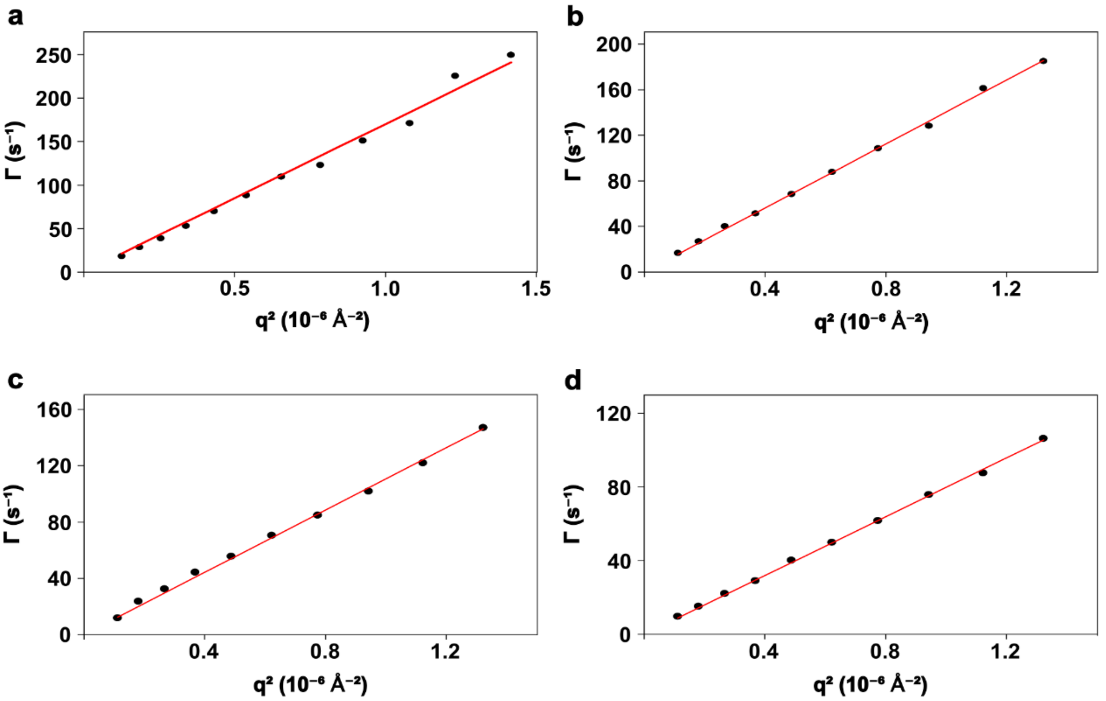

Figure S2. Relaxation rate Γ of g_2 functions versus q^2 of (a) SiO_2-I, (b) SiO_2-II, (c) SiO_2-III and (d) SiO_2-IV.

Table S2. Hydrodynamic diameters (D_H / nm) and free diffusion coefficients obtained via XPCS of SiO_2-I to -V, dispersed in water (at 10 mg/mL) using single exponential model. γ (KWW) is also informed.

Sample	D_0 / μm² s⁻¹ (Single Exponential)	D_H / nm (Single Exponential)	γ ± s.d. (KWW)
**SiO_2-I**	1.6962 ± 0.0285	290.2 ± 4.9	0.99 ± 0.01
**SiO_2-II**	1.3667 ± 0.0089	360.2 ± 2.3	0.99 ± 0.02
**SiO_2-III**	1.0723 ± 0.0123	459.1 ± 5.3	0.97 ± 0.01
**SiO_2-IV**	0.7826 ± 0.0055	629.0 ± 4.4	1.00 ± 0.03
**SiO_2-V**	0.6545 ± 0.0012	752.2 ± 1.4	1.02 ± 0.02

3.2. Effect of media

Subsequently, the dynamic properties of the bare SiO_2-I and PEG-SiO_2 ($D_H \cong 250$ nm) were investigated in more complex environments, following the same protocol employed earlier. These nanoparticles were kept in a fixed concentration of 10 mg/mL and were incubated with bovine serum albumin (BSA / 5 mg/mL) or fetal bovine serum (FBS / 10 mg/mL) media, both dispersed in phosphate buffered saline (PBS / 10 mM) and immediately measured by XPCS (Figure S3(a-f)). All these systems displayed Brownian behavior, regardless of functionalization or media employed (Figure S4(a-f)).

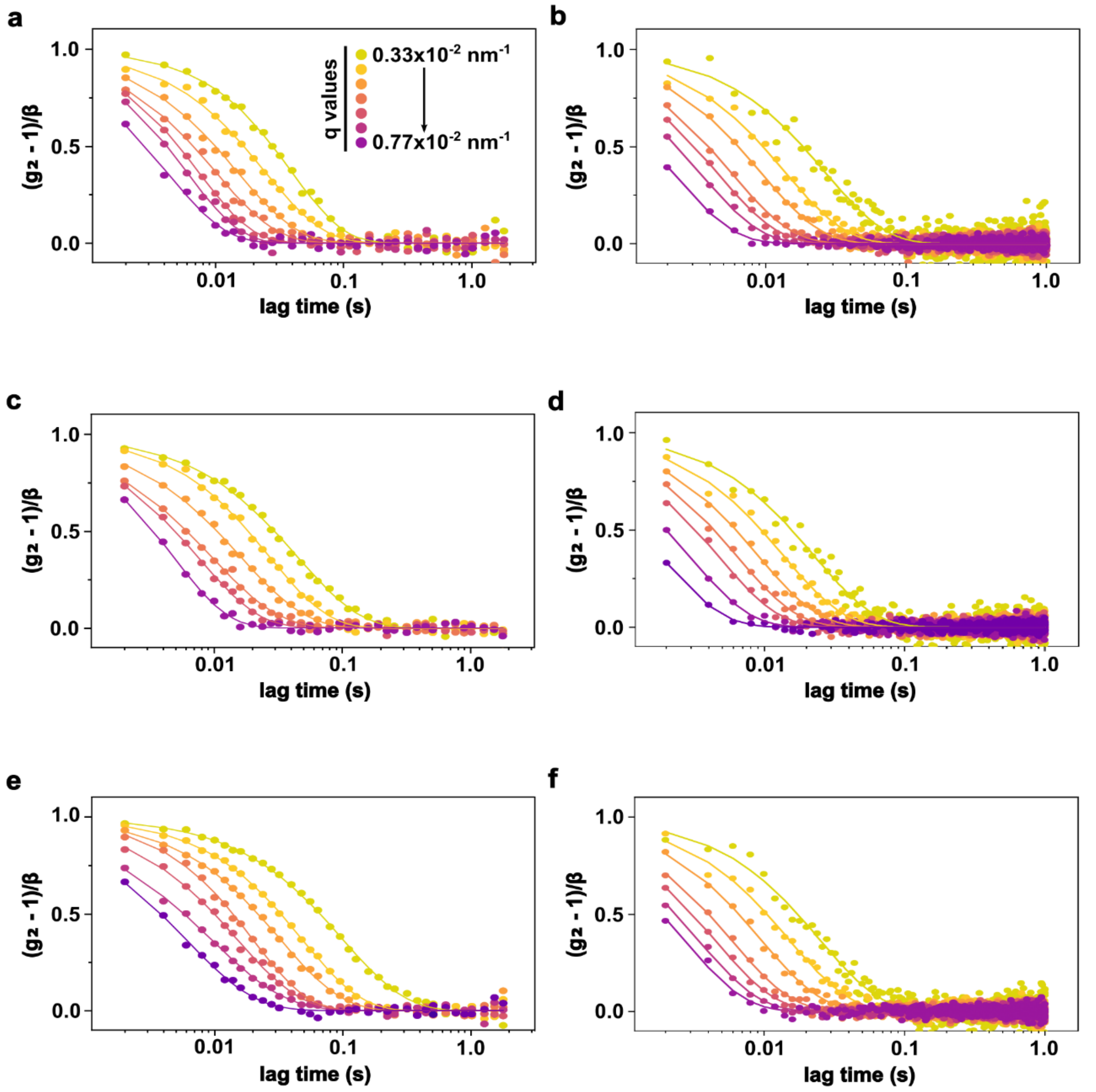

Figure S3. Normalized intensity autocorrelation functions of bare SiO_2-I dispersed in (a) PBS 10 mM, (c) BSA 5 mg/mL, (e) FBS 10%, compared with the same media for PEG-SiO_2 (d) PBS 10 mM (d) BSA 5 mg/mL, (f) FBS 10%.

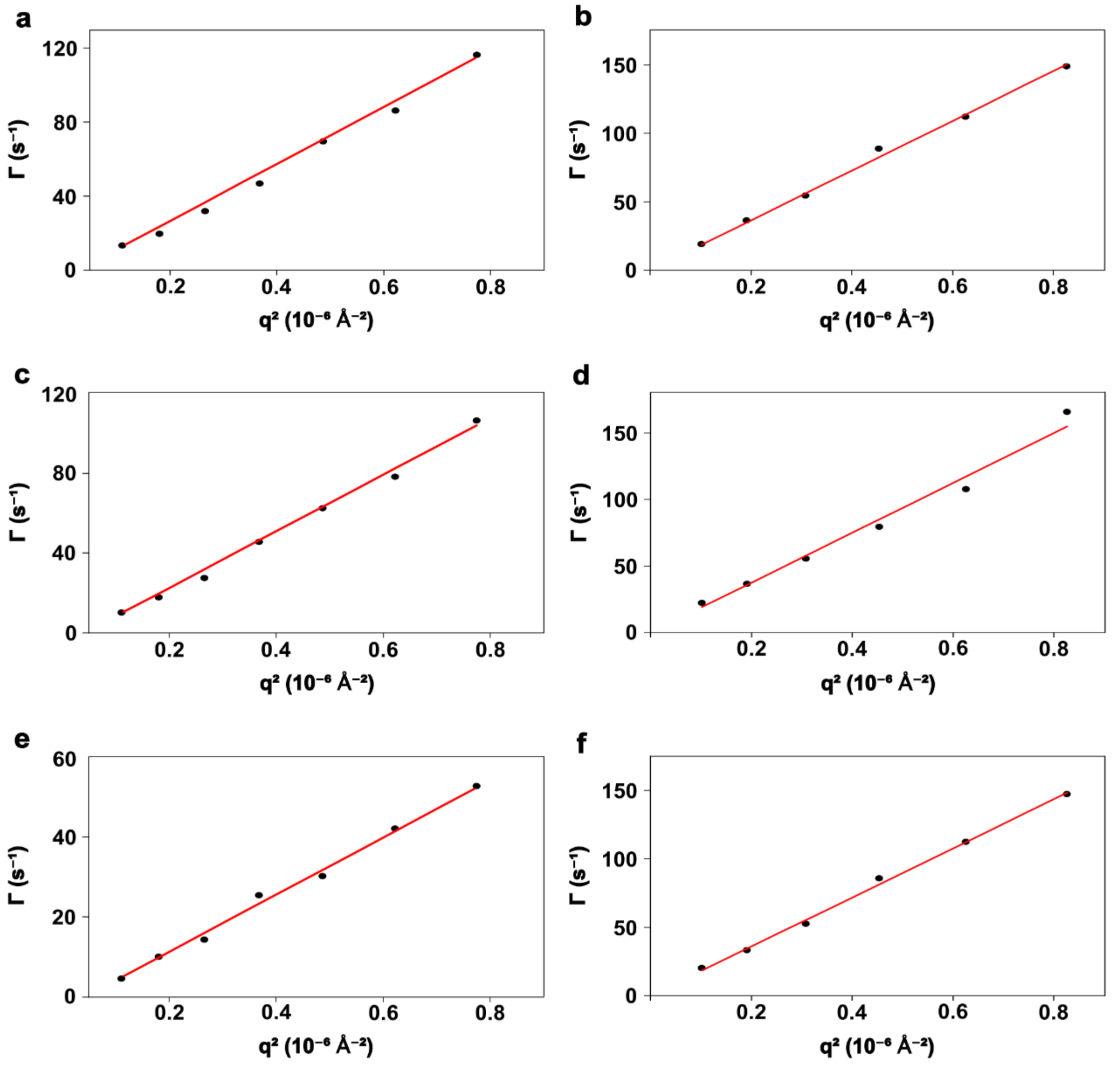

Figure S4. Relaxation rate Γ of g_2 functions versus q^2 of bare SiO_2-I dispersed in (a) PBS 10 mM, (c) BSA 5 mg/mL, (e) FBS 10%, compared with the same media for PEG-SiO_2 (b) PBS 10 mM (d) BSA 5 mg/mL, (f) FBS 10%.

As it was done in the previous study related to effect of the nanoparticles' diameter, the equations S4 and S5 were used to calculate the D_H of SiO_2-I and PEG-SiO_2 in PBS, BSA and FBS. Table S3 summarizes the obtained results employing the single exponential model. The media viscosity values were presented in the Section S5.

Table S3. Average γ exponent, free diffusion coefficients (D_0 / µm² s⁻¹) and hydrodynamic diameters (D_H / nm) obtained via single exponential model of SiO_2-I and PEG-SiO_2, dispersed in PBS (10 mM), BSA (5 mg/mL, dispersed in PBS 10 mM) and FBS (10% v/v, dispersed in PBS 10 mM). $|\Delta D_H| \pm$ standard deviation (s.d.) are also informed, all values were calculated by D_H of the studied medium - D_H in PBS medium. Standard deviation (s.d.) values were calculated by this expression: $\delta_{|\Delta D_H|} = \sqrt{\delta_a^2 + \delta_b^2}$, where δ_a^2 is the s.d. value of D_H of the studied medium and δ_b^2 is the s.d. value of D_H in PBS medium.

Sample	γ ± s.d. (KWW)	D_0 / µm² s⁻¹ (single exponential)	D_H / nm (single exponential)	$\lvert\Delta D_H\rvert$ ± s.d. / nm
**SiO_2-I in PBS**	0.99 ± 0.07	1.6208 ± 0.0577	279.0 ± 9.9	0 ± 14
**SiO_2-I in BSA**	0.99 ± 0.12	1.2888 ± 0.0185	317.0 ± 4.5	38 ± 11
**SiO_2-I in FBS**	0.97 ± 0.05	0.6642 ± 0.0241	581.9 ± 21.1	303 ± 23
**PEG-SiO_2 in PBS**	1.05 ± 0.16	1.8907 ± 0.0706	239.1 ± 8.9	0 ± 14
**PEG-SiO_2 in BSA**	1.03 ± 0.06	1.9067 ± 0.0758	214.1 ± 8.5	25 ± 13
**PEG-SiO_2 in FBS**	1.01 ± 0.01	1.7067 ± 0.03514	225.1 ± 4.7	14 ± 11

3.3. One-way analysis of variance (ANOVA)

To verify if there are statistically significant differences among the $|\Delta D_H|$ in each set (bare SiO_2-I and PEG-SiO_2), we performed a one-way analysis of variance (ANOVA) with Tukey's test, to compare three parametric data, using the software Origin Pro 2023b. Differences were considered significant when $p < 0.05$. For the set of SiO_2-I, we found an F-value of 289.85461 and a p-value < 0.0001, evidencing the significant differences among the $|\Delta D_H|$, with the changes linked to protein corona formation in BSA and aggregation in FBS. On the other hand, for the set of PEG-SiO_2, we found an F-value of 2.90741 and a p-value of 0.13097, indicating no significant difference among $|\Delta D_H|$ values, i.e. PEG-SiO_2 remained nearly unaffected by the presence of proteins (corona-free effect).

3.4. Radiation damage tests

Like other X-ray-based techniques, XPCS poses a risk of radiation damage when applied to biological samples. To mitigate radiation damage, each XPCS time series was collected in a fresh spot. We also performed studies to determine beam-induced changes in structure and/or dynamics. Temporal changes in the scattered intensity during the XPCS measurements can be evidence of structural changes. Figure S5 displays the total scattered intensity *vs.* frame numbers without notable increases or decreases in intensity during the measured time. The absence of beam induced dynamics was verified through the decreasing X-ray dose.

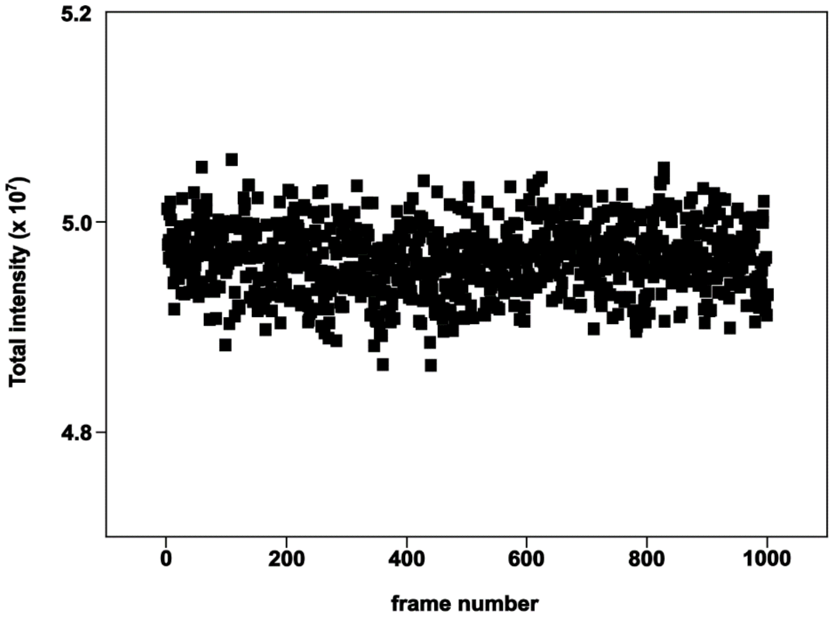

Figure S5. Total intensity *vs.* frame number for the sample SiO_2-I in BSA 5 mg/mL.

The XPCS data was collected at 500 Hz, total exposure time of 2s and within a shorter acquisition time, 1000 Hz, total exposure time of 1s. These curves were superimposed, and they turned out to be practically the same, as well as the relaxation time obtained by single exponential fitting was almost identical. As a representative

example of what was observed for all the samples, we showed the autocorrelation functions g_2 for q = 0.0068 nm^{-1} for the sample SiO_2-I in BSA 5 mg/mL acquired at 500 Hz and 1000 Hz (Figure S6). As expected, the profile obtained at 1000 Hz is noisier as its exposure time is smaller than the 500 Hz, i.e., it has fewer photon counts.

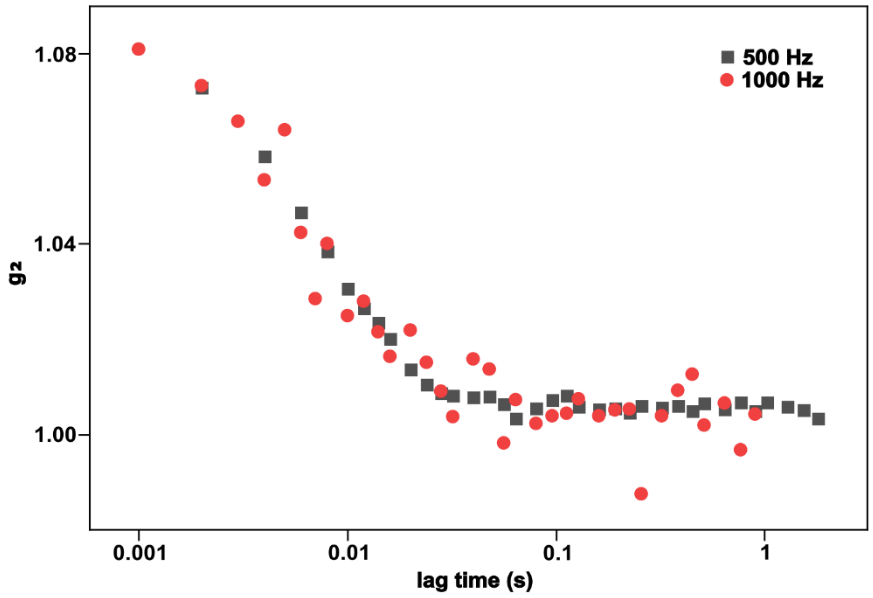

Figure S6. Autocorrelation functions g_2 for q = 0.0068 nm^{-1} for the sample SiO_2-I in BSA 5 mg/mL acquired at the frame rates 500 Hz and 1000 Hz.

Based on these results, we are confident that radiation damage was not an issue in our samples.

Section S4 – STEM images of the bare and PEGylated SiO_2

The spherical morphology of SiO_2-I and PEG-SiO_2 were confirmed by STEM images (Figure S7). Additionally, the average diameter of each sample was measured via ImageJ software by counting 300 independent nanoparticles.

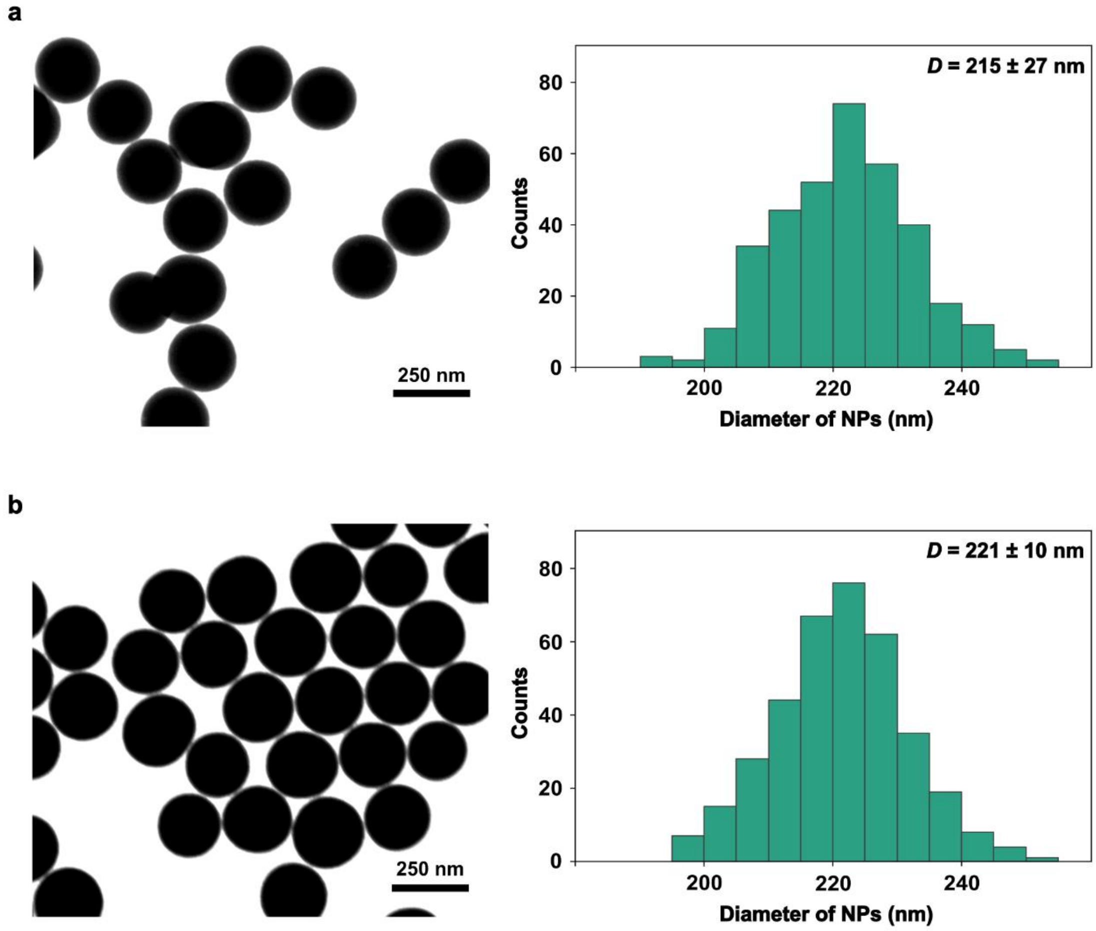

Figure S7. STEM images and average diameter distribution of (a) SiO_2-I and (b) PEG-SiO_2 nanoparticles.

Section S5 – Viscosity measurements

Viscosity measurements were conducted on PBS 10 mM and dispersions of BSA 5 mg/mL and FBS 10% (both dispersed in PBS 10 mM). These measurements were performed using a Haake Mars 60® rheometer, equipped with a CC41DG/Ha double-gap geometry and temperature controller MTMC, maintaining a temperature of 25ºC. Each measurement was conducted in duplicate. A sample volume of 7 mL was utilized for each measurement.

Table S4. Viscosity η values ± standard deviation (s.d.) obtained for PBS 10 mM, BSA 5 mg/mL and FBS 10% (both dispersed in PBS 10 mM)

Sample	η ± s.d. (mPa s)
**PBS 10 mM**	0.966 ± 0.007
**BSA 5 mg/mL in PBS 10 mM**	1.069 ± 0.049
**FBS 10% in PBS 10 mM**	1.137 ± 0.043

Section S6 – Modeling of the SAXS curve of SiO_2-I in FBS

The experimental SAXS curve of SiO_2-I dispersed in media supplemented with FBS 10% v/v, previously showed in the Figure 2c, was fitted as showed below in the Figure S6, using the SASfit software package. Briefly, this SAXS curve was modelled as:

$$I(q) = I_{NP} + I_{agg} + bkg \text{ (Equation S6)}$$

where $I(q)$ is the total scattering intensity and I_{NP} and I_{agg} refer to the scattering contributions of the nanoparticle and the nanoparticle aggregates, respectively. Bkg is a constant that accounts for the background.

Regarding to the I_{NP}, this contribution was modeled as a sphere:

$$I_{sphere}(q, r, \Delta\rho) = k\left[4\pi r^3 \Delta\rho \frac{sinsin\,(qr) - qrcos(qr)}{qr^3}\right]^2 \text{(Equation S7)}$$

where r is the sphere radius and $\Delta\rho$ is the difference between the scattering length density of the sphere and that of the dispersing media. k is a scaling constant that accounts for the number of particles and instrumental parameters.

Concerning to the I_{agg}, mainly manifested at low q region, it was considered by using a power-law decay (with a cut-off constrained to the radius of the nanoparticle (R)) adapted from the Beaucage Unified Model,[12,13] where P refers to a Porod (Power Law) decay exponent of the aggregate.

By applying the described model to fit the experimental SAXS data for SiO_2-I in FBS, we determined the following parameter values: a radius (R) of 110 nm and a Porod exponent (P) of 1.81.

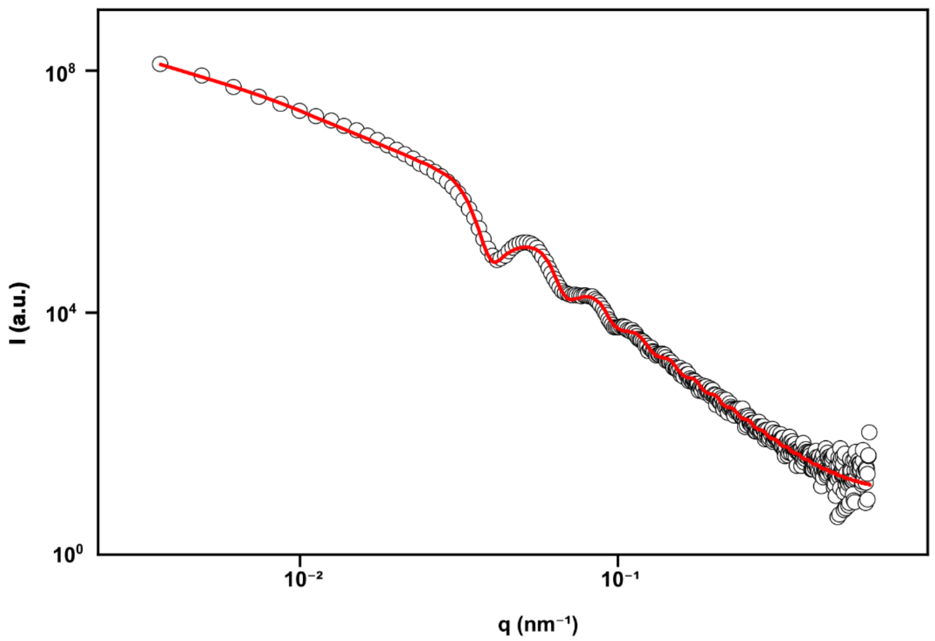

Figure S8. Experimental (open circle) and fitted (red curve) SAXS intensity curves derived from a Beaucage unified model for SiO_2-I dispersed in media supplemented with FBS 10% v/v.

Section S7 - SAXS curves of PEG-SiO_2 in different media

As previously showed in the main text, in the Figure 2c, the SAXS curve of SiO_2-I dispersed in media supplemented with FBS presented a typical aggregation profile, corroborated by the D_H calculated via XPCS. On the other hand, the SAXS profiles of PEG-SiO_2 remained unaffected by the media in which they are dispersed, indicating the high colloidal stability of these systems (Figure S9). These curves were fitted using the Eq. S8 (I_{NP} model as a sphere) and some parameters obtained from this fitting, such as diameter (D) and polydispersity index (PDI), are presented in the Table S5.

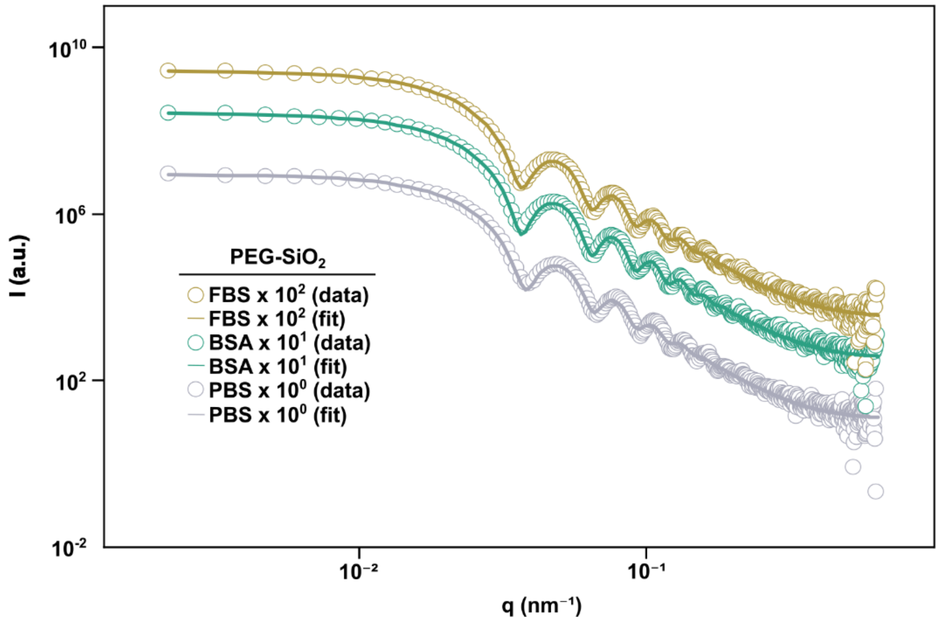

Figure S9. SAXS profiles of PEG-SiO_2 in PBS, BSA and FBS media.

Table S5. Diameter (D) and polydispersity index (PDI) parameters obtained by fitting SAXS experimental data of PEG-SiO_2 in PBS, BSA and FBS media.

System	D (nm)	PDI
**PEG-SiO_2 in PBS**	234	0.06655
**PEG-SiO_2 in BSA**	234	0.055
**PEG-SiO_2 in FBS**	234	0.061754

Section S8 – Theoretical Estimation of the Concentration of Adsorbed BSA

Using simple geometrical considerations, it is possible to estimate the mass corresponding to a monolayer of BSA over SiO_2-I. Considering the nanoparticle diameter obtained by STEM (D_{STEM} = 215 nm), its geometrical specific surface area (SSA_{geo}) can be estimated as:

$$SSA_{geo} = \frac{6000}{D_{STEM} \times \rho_{NP}} \text{ (Equation S8)}$$

where ρ_{NP} is the density of silica (in the nanoparticle form). For Stöber based silica nanoparticles ρ_{NP} ~ 1.9 g/cm^3.[14] Then, it is possible to estimate SSA_{geo} ~ 14.7 m^2/g for SiO_2-I.

BSA has been modeled as an ellipsoid with dimensions of 14 x 4 x 4 nm.[15] Assuming that the adsorbed monolayer is composed of BSA molecules with "side-on" orientation (protein largest axis oriented parallel to nanoparticle surface), the area occupied by one BSA molecule (A_{BSA}) can be estimated by the area of an (projected) ellipse of 14 x 4 nm ($A_{ellipse}$= π x a/2 x b/2) ~ 44 nm^2 or 4.4 x 10^{-17} m^2. Then, the amount of BSA adsorbed (BSA_{ads}) per gram of SiO_2-I can be estimated as:[16]

$$BSA_{ads} = \frac{SSAgeo \times MW_{BSA}}{A_{BSA} \times N_a} \text{ (Equation S9)}$$

where MW_{BSA} is the molecular weight of BSA (~ 66500 Da) and N_a is Avogadro´s number. By replacing with their respective values (using proper unit conversion), an amount of BSA_{ads}~ 0.037 gBSA / gNP is obtained.

In the experiments discussed in the manuscript, using BCA assay, SiO_2-I at 10g/L was exposed to BSA 5 mg/mL. Assuming that nanoparticle surface saturation take place under this condition, and considering the previously calculated $[BSA]_{ads}$, a concentration

of adsorbed BSA ca. 0.37 g/L can be expected. This value is very close to that measured by BCA assay, $[BSA]_{ads}$ = 0.33 g/L (see main text).

References

(1) Otto, F.; Sun, X.; Schulz, F.; Sanchez-Cano, C.; Feliu, N.; Westermeier, F.; Parak, W. J. X-Ray Photon Correlation Spectroscopy Towards Measuring Nanoparticle Diameters in Biological Environments Allowing for the In Situ Analysis of Their Bio-Nano Interface. *Small* **2022**, *18* (37), 2201324. https://doi.org/10.1002/smll.202201324.

(2) Shpyrko, O. G. X-Ray Photon Correlation Spectroscopy. *J Synchrotron Rad* **2014**, *21* (5), 1057–1064. https://doi.org/10.1107/S1600577514018232.

(3) Sutton, M.; Mochrie, S. G. J.; Greytak, T.; Nagler, S. E.; Berman, L. E.; Held, G. A.; Stephenson, G. B. Observation of Speckle by Diffraction with Coherent X-Rays. *Nature* **1991**, *352* (6336), 608–610. https://doi.org/10.1038/352608a0.

(4) Leheny, R. L. XPCS: Nanoscale Motion and Rheology. *Current Opinion in Colloid & Interface Science* **2012**, *17* (1), 3–12. https://doi.org/10.1016/j.cocis.2011.11.002.

(5) Lehmkühler, F.; Roseker, W.; Grübel, G. From Femtoseconds to Hours—Measuring Dynamics over 18 Orders of Magnitude with Coherent X-Rays. *Applied Sciences* **2021**, *11* (13), 6179. https://doi.org/10.3390/app11136179.

(6) Stöber, W.; Fink, A.; Bohn, E. Controlled Growth of Monodisperse Silica Spheres in the Micron Size Range. *Journal of Colloid and Interface Science* **1968**, *26* (1), 62–69. https://doi.org/10.1016/0021-9797(68)90272-5.

(7) Bogush, G. H.; Tracy, M. A.; Zukoski, C. F. Preparation of Monodisperse Silica Particles: Control of Size and Mass Fraction. *Journal of Non-Crystalline Solids* **1988**, *104* (1), 95–106. https://doi.org/10.1016/0022-3093(88)90187-1.

(8) Picco, A. S.; Ferreira, L. F.; Liberato, M. S.; Mondo, G. B.; Cardoso, M. B. Freeze-Drying of Silica Nanoparticles: Redispersibility toward Nanomedicine Applications. *Nanomedicine* **2018**, *13* (2), 179–190. https://doi.org/10.2217/nnm-2017-0280.

(9) Kim, T. G.; An, G. S.; Han, J. S.; Hur, J. U.; Park, B. G.; Choi, S.-C.; Kim, T. G.; An, G. S.; Han, J. S.; Hur, J. U.; Park, B. G.; Choi, S.-C. Synthesis of Size Controlled Spherical Silica Nanoparticles via Sol-Gel Process within Hydrophilic Solvent. *J. Korean Ceram. Soc* **2017**, *54* (1), 49–54. https://doi.org/10.4191/kcers.2017.54.1.10.

(10) Silvano Ramirez, G.; dos Santos Carvalho, J. C.; Gremelmaier Rosa, L.; Miqueles, E. X. *Ssc-Xpcs*, Version 2.0.0; Zenodo: Sirius Scientific Computing, 2024.

(11) Semeraro, E. F.; Möller, J.; Narayanan, T. Multiple-Scattering Effects in SAXS and XPCS Measurements in the Ultra-Small-Angle Region. *Journal of Applied Crystallography* **2018**, *51* (3), 706–713. https://doi.org/10.1107/S160057671800417X.

(12) Beaucage, G. Approximations Leading to a Unified Exponential/Power-Law Approach to Small-Angle Scattering. *Journal of Applied Crystallography* **1995**, *28* (6), 717–728. https://doi.org/10.1107/S0021889895005292.

(13) Beaucage, G. Small-Angle Scattering from Polymeric Mass Fractals of Arbitrary Mass-Fractal Dimension. *Journal of Applied Crystallography* **1996**, *29* (2), 134–146. https://doi.org/10.1107/S0021889895011605.

(14) Labrosse, A.; Burneau, A. Characterization of Porosity of Ammonia Catalysed Alkoxysilane Silica. *Journal of Non-Crystalline Solids* **1997**, *221* (2), 107–124. https://doi.org/10.1016/S0022-3093(97)00414-6.

(15) T. Peters. *All about Albumin : Biochemistry, Genetics, and Medical Applications*; Academic Press, 1996.

(16) Picco, A. S.; Mondo, G. B.; Ferreira, L. F.; Souza, E. E. de; Peroni, L. A.; Cardoso, M. B. Protein Corona Meets Freeze-Drying: Overcoming the Challenges of Colloidal Stability, Toxicity, and Opsonin Adsorption. *Nanoscale* **2021**, *13* (2), 753–762. https://doi.org/10.1039/D0NR06040B.
